# Improvement of glucose metabolism in pregnant women through probiotic supplementation depends on gestational diabetes status: meta-analysis

**DOI:** 10.1038/s41598-020-74773-8

**Published:** 2020-10-20

**Authors:** Karolina Łagowska, Anna M. Malinowska, Bogna Zawieja, Emilia Zawieja

**Affiliations:** 1 Department of Human Nutrition and Dietetics, Poznań University of Life Sciences, Wojska Polskiego 31, 60-624 Poznań, Poland; 2 Department of Mathematical and Statistical Methods, Poznań University of Life Sciences, 60-637 Poznań, Poland

**Keywords:** Diseases, Health care

## Abstract

The aim of this study was to assess the effects of probiotic and synbiotic supplementation on glucose metabolism in pregnant women using data from randomized controlled trials. Furthermore, this meta-analysis examines whether the observed effects depend on the presence or absence of gestational diabetes mellitus (GDM), and if the effect is dependent on the type of supplement used (probiotic or synbiotic). We performed a literature search of databases (Medline, Scopus, Web of Knowledge, and Cochrane Library) and identified all relevant randomized controlled trials (RCTs) published prior to May 2019. We compared the effects of probiotic supplementation with the administration of placebos in pregnant women with and without GDM. The systematic review and meta-analysis protocol were registered in the International Prospective Register of Systematic Reviews as number CRD 42019111467. 1119 study participants from 15 selected studies were included. The participants in four studies did not have GDM (being recruited to the study before week 20 of pregnancy) and the participants in the rest of the studies were diagnosed with GDM between weeks 24 and 28 of gestation. The meta-analysis showed that supplementation lowers serum glucose, insulin levels, and HOMA-IR index, but only in pregnant women with GDM. Moreover, both probiotics and synbiotics lower serum insulin level and HOMA-IR index, but the glucose lowering effect is specific only to probiotics and not synbiotics. Probiotic supplementation may improve glucose metabolism in pregnant women with GDM. There is a need for more RCT studies with larger groups to better estimate this effect.

## Introduction

Gestational diabetes mellitus (GDM), glucose intolerance, and insulin resistance during pregnancy are medical problems in which prevalence is increasing worldwide. Untreated GDM increases the risk of miscarriage, preterm birth, preeclampsia, induction of labor and caesarean section, and macrosomia. GDM also increases the risk of later maternal and child obesity and type-2 diabetes mellitus^1^.

Lifestyle interventions, including diet and physical activity have been demonstrated to reduce the risk of obesity and diabetes mellitus, and also to positively affect anthropometric and biochemical parameters of both mother and child. Recently, it has also been shown that gut microbiota dysbiosis is associated with obesity and several metabolic diseases, including insulin resistance and type-2 diabetes^2,3^. Probiotics are live microorganisms which, when administered in appropriate amounts, may confer a health benefit on the host^3,4^. An early Finnish study showed that the intake of a probiotic supplement containing *Lactobacillus rhamnosus GG* and *Bifidobacterium lactisBb12*, taken from the first trimester of pregnancy, reduced the prevalence of GDM from 34 to 13%^5^.

Prebiotics—non-digestible selectively fermented dietary fiber—are also receiving more attention because of their desirable characteristics^6^. The health benefits of prebiotics result largely from the fact that they are substrates for the production of short-chain fatty acids (SCFA), such as acetate, propionate, butyrate, and lactate, which reduce pH in the lumen, possibly preventing colonization by acid-sensitive enteropathogens^6^. Acetate production also contributes to the production of butyrate, which is a primary substrate for colonocytes, thus contributing to epithelial integrity^7,8^. SCFAs directly modulate host health through different mechanisms related to gut barrier function, immunomodulation, glucose homeostasis, appetite regulation, and obesity^9^. There are many studies where probiotics and prebiotics are administered together as synbiotics^10,11^. The results of recent meta-analysis suggested that synbiotic supplementation may help improve biomarkers of inflammation and oxidative stress in diabetic patients, glucose homeostasis parameters, as well as hormonal and inflammatory indices in diabetes patients and in women with polycystic ovary syndrome^12–14^.

The efficacy of any dietary supplement, including probiotics, prebiotics, and synbiotics may depend on the individual characteristics of the individual, such as genotype, presence or absence of various diseases (which may alter metabolism), intake of supplements and drugs, and other factors^15–18^. Moreover, gut microbiota composition may affect the bioavailability of nutrients, and consequently the nutritional status and metabolism of the host^19,20^.

Although the last two years has seen the publication of five meta-analyses and systematic reviews aimed at determining the effect of modifications of the gut microbiota composition on glucose metabolism in pregnant women, three of them were restricted only to pregnant women with GDM^21–23^. Although the other two meta-analyses included women both with and without GDM, synbiotic supplementation was either not included or the effects of probiotics and synbiotics were not considered separately^24,25^. Moreover, new studies have emerged since the publication of these meta-analyses^26–28^.

The aim of this study was therefore to assess the effects of probiotic and synbiotic supplementation on glucose metabolism in pregnant women using the data available from randomized controlled trials. Furthermore, this meta-analysis examines whether the effects observed depend on the presence or absence of GDM—on whether probiotics or synbiotics could be useful in the prevention or treatment of GDM–and whether the efficacy depends on the type of supplement (probiotic or synbiotic).

## Material and methods

### PRISMA guideline and the PICO principle

This systematic review and meta-analysis protocol were registered in the International Prospective Register of Systematic Reviews (Prospero) as number CRD42019111467^29^. The Preferred Reporting Items for Systematic Reviews and Meta-Analyses (PRISMA) guidelines^30^ were consulted throughout the execution of the review. Participants, interventions, comparators, outcomes, study design (PICOS) criteria were defined as in Table 1.

**Table 1:** PICOS criteria for inclusion of studies.

PICOS criteria	Definition of criteria for studies
Participants	Pregnant women (aged 18–45 years)
Intervention	Oral supplementation of probiotic or synbiotic
Comparator	Systematic review: control/placebo Meta-analysis: control/placebo
Outcomes	Primary outcome: glucose, insulin, HOMA-IR
Study design	Systematic review: randomized controlled trials (RCTs) Meta-analysis: randomized controlled trials (RCTs)

*RCT* randomized controlled trial.

### Literature sources, search strategy, and selection criteria

A systematic review of the literature was undertaken from April 2018 to May 2019 using the Cochrane Library, PubMed/Medline, Scopus, and Web of Science. No role in the decision to publish, manuscript preparation, analysis of the data, collection of the data, or design of the study was played by the funding body. The authors did not possess any competing interests. The databases were searched using the following key words and their varying combinations: “probiotics” OR “bacteria” AND “supplementation” OR “supplement” AND “pregnancy” OR “gestation” OR “pregnant” OR “gestational diabetes” OR “GDM” AND “glucose” OR “insulin” OR “HbA1c” OR “glycosylated hemoglobin A1c” OR “glycemic control” OR “metabolism “OR” insulin resistant” OR “oral glucose test” OR “OGTT” OR “homeostasis model assessment” OR “HOMA-IR”.

Three approaches were used to the search:

1. four nutrition and dietetics databases were searched for appropriate articles;
2. references to similar works were extracted from the articles, and
3. the literature cited in the reviews was searched.

We also consulted with a reference librarian with the aim of verifying our database sources and research procedures. We only included randomized controlled trials (RCTs) that met the following criteria:

1. they were studies of the effects of probiotics and/or synbiotics on carbohydrate metabolism during pregnancy;
2. they measured glucose parameters;
3. they had any date of publication;
4. pregnant women aged 18–45 were the primary study population;
5. they presented data on fasting glucose, insulin level, and HOMA-IR from both before and after intervention;
6. they presented sufficient data for analysis.

Studies were excluded if:

1. the clinical outcomes of pregnancy could not be ascertained;
2. the women were diagnosed with type-2 diabetes mellitus before pregnancy;
3. they were observational or preclinical in design;
4. they were reviews, conference abstracts, case reports, editorials, or book chapters;
5. they were not in English.

### Extraction and analysis of data

Data was independently extracted by two reviewers who applied the inclusion and exclusion criteria. Titles, abstracts, and full texts of publications were progressively examined. Where a full text version was not available, the authors were contacted directly. The nine-point Newcastle–Ottawa scoring system was used to assess the quality of each study. The highest score achievable was 9, and we further considered a study to be high-quality if it received 7 or more points^31^. Two authors each independently assessed the eligibility of every study found in the databases, extracting the necessary data from each. During this process, we attempted to contact the study authors in order to gain further information that had not been published. Any disagreements that arose were solved through arbitration or consensus. The data we extracted from each study included the name of the journal, country, publication year, name of the first author, sample size, study design, a full description of the participants and their age, the interventions used (including frequency and type), the control interventions, and the main outcomes (such as HOMA-IR index, fasting insulin, and fasting glucose).

### Bias assessment

We made use of the Cochrane risk of bias assessment tool in order to judge the methodological quality of each trial, with the aim of evaluating the performance and methods of randomization, the extent of blinding (whether it affected data collectors, data analysis, outcome assessors, and participants), allocation concealment, incomplete outcome data, selective reporting, and other possible sources of bias. In line with the Cochrane handbook’s criteria for judging bias risk, each study was stated to have a high, low, or unclear risk of bias^32^.

### Statistical analysis

Statistical analyses were carried out using the R package. The therapeutic effect of probiotic supplementation on glucose and insulin metabolism in pregnant women in randomized controlled trials with placebo was estimated using the standardized mean difference (SMD) with a 95% confidence intervals (CI). The effect size was estimated as pretest–posttest–control using the pooled pretest *SD* method described by Morris et al.^33^. The correlation coefficients were calculated from SD differences or from *p*-value differences; if missing this coefficient was taken as 0.5^34^. The random-effects (RE) model was used. The statistical analysis of the overall SMD was evaluated using the Z-test. Heterogeneity across studies was evaluated by Cochran’s *Q*-statistic (*p* < 0.1 implying significant difference) and the *I*^2^-statistic (*I*^2^ = 0% meaning no heterogeneity; *I*^2^ = 100% meaning maximal heterogeneity). Outliers were detected using the studentized residuals *r*_*i*_ and Cook’s statistic *D*_*i*_. Publication bias was assessed by a funnel plot and tested using the Egger method^35^. The trim and fill method was used to assess the number of missing studies on the one side of the funnel plot^36,37^. All statistical tests were two-sided, and *p* values < 0.05 were considered statistically significant.

## Results

### Search results

**Figure 1:**
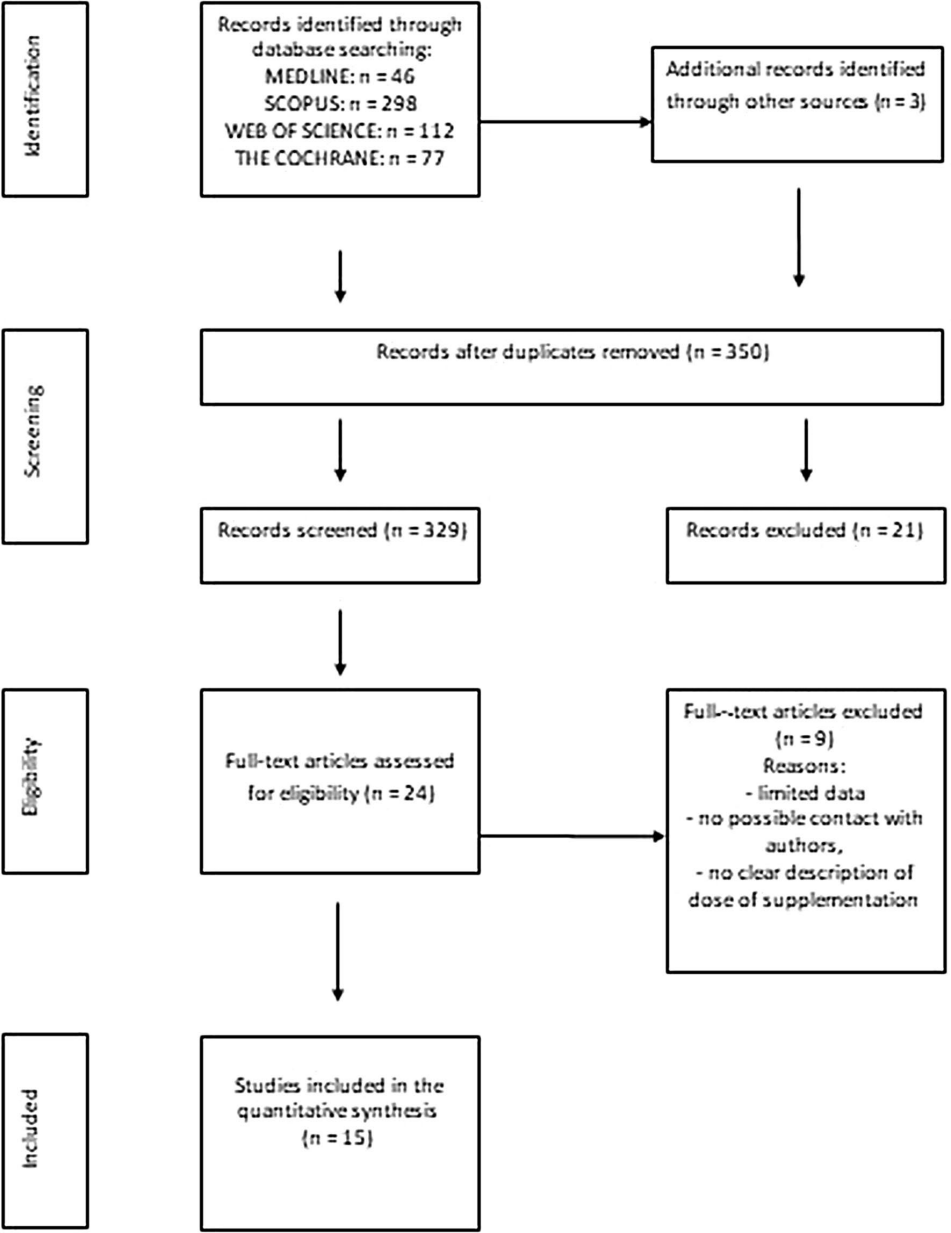
Flow diagram of the literature search procedure.

A flow chart showing the study extraction is presented in Fig. 1. Throughout initial search strategy, we identified 533 articles, and following the further analysis of the titles and abstracts section, 24 publications were selected for full-text review. Duplicate articles, publications with insufficient data, and publications where the authors could not be contacted were ruled out. Finally, 15 RCTs met the inclusion criteria and were included in the final meta-analysis.

### Population and study characteristics

The risk of bias evaluated by the Cochrane Collaboration tools for RCT is presented in Table 2. Articles that were included in this analysis appropriately reported randomization and dropout rates. Moreover, the generation of allocation sequences was adequate and explicit mention of intention-to-treat was evident in fourteen out of fifteen manuscripts. Most of the studies used supplements in capsules. Yogurt enriched with probiotics was ingested by study participants in two of the studies^27,38^, and synbiotic foods were used in another^39^. In nine studies, probiotic capsules were given daily^28,40–47^, while a synbiotic capsule was ingested daily in the remaining three^28,48,50^.

**Table 2:** Characteristics of the included randomized controlled trial ECRs (Cochrane Collaboration).

	Random sequence generation	Allocation concealment	Blinding participants/personnel	Blinding outcome assessment	Incomplete outcome data	Selective reporting
Asemi et al. (2013)^38^	↓	?	?	?	↓	?
Jamilian et al. (2016)^47^	↓	↓	↓	↓	↓	↓
Lindsay et al. (2014)^40^	↓	↓	↓	↓	↓	↓
Taghizadeh et al. (2014)^39^	↓	↓	↓	↓	↓	↓
Ahmadi et al. (2016)^26^	↓	↓	↓	↓	↓	↓
Asgharian et al. (2018)^27^	↓	↓	↓	↓	↓	↓
Babadi et al. (2018)^28^	↓	↓	↓	↓	↓	↓
Badehnoosh et al. (2017)^42^	↓	↓	↓	↓	↓	↓
Dolatkhah et al. (2015)^43^	↓	↓	↓	↓	↓	↓
Jafarnejad et al. (2016)^44^	↓	↓	↓	↓	↓	↓
Jamilian et al. (2019)	↓	↓	↓	↓	↓	↓
Karamali et al. (2016)^45^	↓	↓	↓	↓	↓	↓
Kijmanawat et al. (2018)^46^	↓	↓	↓	↓	↓	↓
Lindsay et al. (2015)	↓	↓	↓	↓	↓	↓
Nabhani et al. (2018)^49^	↓	↓	↓	↓	↓	↓

↑ high risk; ↓ low risk; ? unclear.

The basic characteristics, number of subjects, experiment duration, type of intervention, and carbohydrate metabolism parameters are presented in Tables 3 and 4. A total of 1119 study participants were included from fifteen selected studies. The participants of four studies (n = 320) did not have GDM (being recruited to the study before week 20 of pregnancy), while the participants in the rest of the studies (n = 799) were diagnosed with GDM between 24 and 28 week of gestation. The date of publication of the selected articles was not limited, but all articles included in this article were published after 2012.

**Table 3:** Characteristics of studies and population (n = 1119).

Study	Country	Intervention	Type	Manufacturer	Time of intervention (weeks)	Age (years) Mean (SD)	Week of pregnancy at the start of intervention	GDM present
Asemi et al. (2013)^38^	Iran	SG (n = 37) Yoghurt with *Streptococcus thermophilus* and *bulgaricus* enriched with probiotic culture of two strains of lactobacillus (*L. acidophilus LA5*) and Bifidobacteria (*B. animalis BB12*) with a total of min 10^7^ CFU (200 g per day) PG (n = 33) Conventional yoghurt with *Streptococcus thermophilus* and *bulgaricus* (200 g per day)	Probiotic yoghurt	N/A	9	18–30^a^	Third trimester	No
Jamilian et al. (2016)^47^	Iran	SG (n = 30) *Lactobacillus acidophilus*, *Lactobacillus casei*, *Bifidobacterium bifidum* + 0.8 g inulin (1 capsule per day) PG (n = 30) N/A (1 capsule per day)	Synbiotic capsules	Tak Gen Zist Pharmaceutical Company, Tehran, Iran	12	18–37^a^	9 week of gestation	No
Lindsay et al. (2014)^40^	Ireland	SG (n = 63) 100 mg of 10^9^ of *Lactobacillus salivarius UCC118* (1 capsule per day) PG (n = 75) N/A	Probiotic capsules	Alimentary Health, Cork, Ireland	4	SG: 31.4 (5.0) PG: 31.0 (5.2)	24–28 week of gestation	No
Taghizadeh et al. (2014)^39^	Iran	SG (n = 26) Synbiotic food (18 g/per day) consisting of a probiotic *Lactobacillus sporogenes* (1 × 10^7^ CFU) + 0.04 g inulin as prebiotic with 0.38 g isomalt, 0.36 g sorbitol, and 0.05 g stevia as sweetener per 1 g (18 g/per day) PG (n = 26) Control food (18 g/per day) (the same food without probiotic bacteria and inulin)	Synbiotic foods	SekkehGaz Company, Isfahan, Iran	9	SG: 26.4 (6.3) PG: 29.0 (4.6)	Third trimester	No
Ahmadi et al. (2016)^26^	Iran	SG (n = 35) *Lactobacillus acidophilus*, *Lactobacillus casei*, *Bifidobacterium bifidum* (1 × 10^9^ CFU/g) + 0.8 g inulin (1 capsule per day) PG (n = 35) capsules containing starch without bacteria and inulin (1 capsule per day)	Synbiotic capsules	Tak Gen Zist Pharmaceutical Company, Tehran, Iran	6	18–40^a^	24–28 week of gestation	Yes
Asgharian et al. (2018)^27^	Iran	SG (n = 37) 100 g yoghurt with *Streptococcus thermophilus* and *Lactobacillus delbrueckii* subsp. *bulgaricus* 10^7^ CFU/g enriched with 5 × 10^8^ CFU/g *Lactobacillus acidophilus* and *Bifidobacterium lactis* PG (n = 33) 100 g of conventional yoghurt with *Streptococcus thermophilus* and *Lactobacillus delbrueckii* subsp. *bulgaricus* 10^7^ CFU/g	Probiotic yoghurt	Pegah Dairy Factory, Tabriz, Iran	from 24 weeks of gestation until delivery	SG: 29.5 (6.2) PG: 29.4 (5.5)	24–28 week of gestation	Yes
Babadiet al. (2018)^28^	Iran	SG (n = 24) *Lactobacillus acidophilus, Lactobacillus casei*, *Bifidobacterium bifidum*, *Lactobacillus fermentum* (2 × 10^9^ CFU/g each) PG (n = 24) Corn starch	Probiotic capsules	LactoCare Zist Takhmir Pharmaceutical Company, Tehran, Iran; Barij Pharmaceutical Company, Kashan, Iran	6 weeks	SG: 28.8 (4.3) PG: 29.0 (4.2)	24–28 week of gestation	Yes
Badehnoosh et al. (2017)^42^	Iran	SG (n = 30) *Lactobacillus acidophilus*, *Lactobacillus casei* and *Bifidobacterium bifidum* (2 × 10^9^ CFU/g each) (1 capsule per day) PG (n = 30) capsules containing starch (1 capsule per day)	Probiotic capsules	Tak Gen Zist Pharmaceutical Company, Tehran, Iran	6	SG: 27.8 (3.7) PG: 28.8 (5.4)	24–28 week of gestation	Yes
Dolatkhah et al. (2015)^43^	Turkey	SG (n = 27) 4 × 10^9^of *Lactobacillus acidophilus LA-5, Bifidobacterium BB-12, Streptococcus thermophilus STY-31*, *Lactobacillus delbrueckii* subsp. *bulgaricus LBY-27* (1 capsule per day) PG (n = 29) N/A	Probiotic capsules	CHR Hansen, Denmark	8	SG: 28.1 (6.2) PG: 26.5 (5.2)	24–28 week of gestation	Yes
Jafarnejad et al. (2016)^44^	Iran	SG (n = 41) 112.5 × 10^9^ CFU of *Streptococcus thermophilus*, *Bifidobacterium breve*, *Bifidobacterium longum*, *Bifidobacterium infantis*, *Lactobacillus acidophilus*, *Lactobacillus plantarum*, *Lactobacillus paracasei*, *Lactobacillus delbrueckii* subsp*. bulgaricus* (1 capsule per day) PG (n = 41) Capsules containing 40 mg microcrystalline cellulose	Probiotic capsules	N/A	8	SG: 32.4 (3.1) PG: 31.9 (4.0)	24–28 week of gestation	Yes
Jamilian et al. (2019)^48^	Iran	SG (n = 29) 8 × 10^9^ CFU/g *Lactobacillus acidophilus*, *Bifidobacterium bifidum*, *Lactobacillus reuteri*, *Lactobacillus fermentum* (2 × 10^9^ each) PG (n = 28) Paraffin and starch	Probiotic capsules	LactoCare ZistTakhmir Pharmaceutical Company, Tehran, Iran; Barij Pharmaceutical Company, Kashan, Iran	6	PG: 31.2 (5.9) SG: 29.9 (3.7)	24–28 week of gestation	Yes
Karamali et al. (2016)^45^	Iran	SG (n = 30) *Lactobacillus acidophilus* (2 × 10^9^ CFU/g), *Lactobacillus casei* (2 × 10^9^ CFU/g) *Bifidobacterium bifidum* (2 × 10^9^ CFU/g)(1 capsule per day) PG (n = 30) Capsules containing starch without bacteria	Probiotic capsules	Lactofem ZistTakhmir Pharmaceutical Company, Tehran, Iran	6	18–40^a^	24–28 week of gestation	Yes
Kijmanawat et al. (2018)^46^	Thailand	SG (n = 29) *Bifidobacterium bifidum* (10^6^ CFU), *Lactobacillus acidophilus* (10^6^ CFU), 1 capsule per day PG (n = 28) Gelatin	Probiotic capsules	Infloran Laboratorio, Farmaceutico SIT, Mede, Italy, imported by DKSH, Bangkok, Thailand	4	PG: 32.5 (5.02) SG: 30.7 (5.05)	24–28 week of gestation	Yes
Lindsay et al. (2015)^41^	Ireland	SG (n = 75) 100 mg *Lactobacillus salivarius UCC118* (10^9^ CFU), 1 capsule per day PG (n = 74) N/A	Probiotic capsules	Alimentary Health, Cork, Ireland	4–6	PG: 33.5 (5.0) SG: 32.6 (4.5)	< 34 week of gestation	Yes
Nabhani et al. (2018)^49^	Iran	SG (n = 45) 500 mg *Lactobacillus* probiotic strains consisting of *Lactobacillus acidophilus* (5 × 10^10^ CFU/g), *Lactobacillus plantarum* (1.5 × 10^10^ CFU/g), *Lactobacillus fermentum* (7 × 10^9^ CFU/g), *Lactobacillus gasseri* (2 × 10^10^ CFU/g) and 38.5 mg FOS as prebiotic substance, 1 capsule per day PG (n = 45) Capsules contained lactose (300 mg), magnesium stearate, talc, colloidal silicon dioxide (5.5 mg each), 1 capsule per day	Synbiotic capsules	Lactofem ZistTakhmir Pharmaceutical Company, Tehran, Iran	6	PG: 29.4 (5.8) SG: 30.3 (5.6)	24–28 week of gestation	Yes

*N/A* not available; *SG* supplemented group; *PG* placebo group.

^a^Age range of participants.

**Table 4:** Mean fasting glucose concentration (mg/dl), fasting insulin level (µlU/L), and value of HOMA-IR index before and after supplementation with probiotics or synbiotics in the supplemented and placebo groups.

Study	Supplementation protocol	Fasting glucose (mg/dL)				Fasting insulin (µIU/mL)				HOMA-IR			
		Before		After		Before		After		Before		After	
		Mean	SD	Mean	SD	Mean	SD	Mean	SD	Mean	SD	Mean	SD
Asemi et al. (2013)^38^	SG: Yoghurt with *Streptococcus thermophiles* and *bulgaricus* enriched with probiotic culture of two strains of lactobacilli (*L. acidophilus LA5*) and Bifidobacteria (*B. animalis BB12*) PG: Yoghurt with *Streptococcus thermophiles* and *bulgaricus*	95.6 91.6	4.0 4.3	74.3* 75.4	2.3 2.1	8.8 6.9	1.0 1.1	10.0 11.9	1.2 1.2	2.1 1.5	0.3 2.2	1.9 1.0	0.2 1.2
Jamilian et al. (2016)^47^	SG: *Lactobacillus acidophilus*, *Lactobacillus casei*, *Bifidobacterium bifidum* + 0.8 g inulin PG: no data	81.6 83.0	7.9 6.7	80.3 82.8	8.7 6.9	11.1 12.8	5.3 9.5	9.6* 14.1	4.7 9.3	2.3 2.6	1.1 2.0	2.0* 2.9	1.0 1.9
Lindsay et al. (2014)^40^	SG: 10^9^ of *Lactobacillus salivarius UCC118* PG: no data	84.51 85.78	7.75 8.47	82.89 84.51	7.21 8.29	13.85 16.67	4.62 7.85	15.36 16.88	6.35 5.75	2.94 3.54	1.17 1.91	3.26 3.53	1.58 1.32
Taghizadeh et al. (2014)^39^	SG: Synbiotic food consisting of a probiotic *Lactobacillus sporogenes* (1 × 10^7^ CFU) + 0.04 g inulin as prebiotic with 0.38 g isomalt, 0.36 g sorbitol, and 0.05 g stevia as sweetener per 1 g PG: Control food (the same food without probiotic bacteria and inulin)	65.26 72.80	22.93 10.37	62.88 69.92	17.81 14.81	11.79 9.40	8.61 7.89	11.53* 15.74*	6.56 15.19	1.95 1.63	1.73 1.29	1.82* 2.76	1.32 3.10
Ahmadi et al. (2016)^26^	SG: *Lactobacillus acidophilus*, *Lactobacillus casei*, *Bifidobacterium bifidum* (1 × 10^9^ CFU/g) + 0.8 g inulin PG: capsules containing starch without bacteria and insulin	96.2 92.1	8.0 9.2	94.5 93.5	8.4 10.3	13.1 13.3	7.1 5.4	11.6* 18.1	3.8 12.6	3.1 3.1	1.7 1.4	2.7* 4.2	1.0 2.8
Asgharian et al. (2018)^27^	SG: (n = 37) 100 g of yoghurt with *Streptococcus thermophilus* and *Lactobacillus delbrueckii* subsp. *bulgaricus* 10^7^ CFU/g enriched with 5 × 10^8^ CFU/g *Lactobacillus acidophilus* and *Bifidobacterium lactis* PG: (n = 33) 100 g of conventional yoghurt with *Streptococcus thermophilus and Lactobacillus delbrueckii* subsp. *bulgaricus* 10^7^ CFU/g	75.5 74.1	7.2 7.0	74.8 77.9	7.4 11.2	N/A	N/A	N/A	N/A	N/A	N/A	N/A	N/A
Babadi et al. (2018)^28^	SG: (n = 24) *Lactobacillus acidophilus, Lactobacillus casei*, *Bifidobacterium bifidum*, *Lactobacillus fermentum* (2 × 10^9^ CFU/g each) PG: (n = 24) Corn starch	92.2 90.3	11.2 6.9	89.2* 91.3	8.9 8.7	12.0 11.8	2.3 2.3	10.5* 12.7*	2.3 3.8	2.7 2.6	0.6 0.5	2.3* 2.9	0.5 1.1
Badehnoosh et al. (2017)^42^	SG: capsules: *Lactobacillus acidophilus*, *Lactobacillus casei*, and *Bifidobacterium bifidum* (2 × 10^9^ CFU/g each) PG: capsules containing starch	94.0 91.8	5.5 7.5	88.7* 91.8	7.1 8.7	N/A	N/A	N/A	N/A	N/A	N/A	N/A	N/A
Dolatkhah et al. (2015)^43^	SG: 4 × 10^0^ of *Lactobacillus acidophilus LA-5*, *Bifidobacterium BB-12*, *Streptococcus thermophilus STY-31*, *Lactobacillus delbrueckii bulgaricus LBY-27* PG: no data	103.65 100.89	1.34 1.52	88.37* 93.59*	2.05 3.61	5.95 5.60	0.50 0.37	5.15 6.12	0.41 0.5	1.52 1.38	0.12 0.08	1.11* 1.40	0.09 0.11
Jafarnejad et al. (2016)^44^	SG: 112.5 × 109 CFU: *Streptococcus thermophilus*, *Bifidobacterium breve*, *Bifidobacterium longum*, *Bifidobacterium infantis*, *Lactobacillus acidophilus*, *Lactobacillus plantarum*, *Lactobacillus paracasei*, *Lactobacillus delbrueckii* subsp*. bulgaricus* PG: Capsules containing 40 mg microcrystalline cellulose	91.6 93.7	4.3 3.1	89.3 88.9	3.4 4.4	19.1 18.7	4.2 5.8	16.6* 22.3*	5.9 4.9	4.2 4.4	1.2 1.3	3.7* 4.9*	1.5 1.2
Jamilian et al. (2019)	SG: (n = 29) 8 × 10^9^ CFU/g *Lactobacillus acidophilus*, *Bifidobacterium bifidum*, *Lactobacillus reuteri*, *Lactobacillus fermentum* (each 2 × 10^9^) PG: (n = 28) Paraffin and starch	96.6 94.1	3.4 6.1	86.5 93.0	7.6 7.9	13.1 13.6	7.7 2.5	11.7 13.4	6.6 2.9	3.1 3.1	1.9 0.6	2.5 3.1	1.5 0.8
Karamali et al. (2016)^45^	SG: *Lactobacillus acidophilus* (2 × 10^9^ CFU/g), *Lactobacillus casei* (2 × 10^9^ CFU/g) *Bifidobacterium bifidum* (2 × 10^9^ CFU/g) PG: Capsules containing starch without bacteria	96.9 91.1	7.6 9.6	87.7* 92.2	7.1 10.5	12.0 13.2	4.8 5.5	11.2* 17.8*	4.4 12.3	2.9 3.0	1.2 1.4	2.5* 4.1*	1.0 2.7
Kijmanawat et al. (2018)^46^	SG: *Bifidobacterium bifidum* (10^6^ CFU), *Lactobacillus acidophilus* (10^6^ CFU) PG: gelatin	82.96 83.68	6.7 8.3	83.92* 88.31	6.48 8.74	8.77 6.76	4.56 3.98	9.88* 10.53	4.15 5.33	1.82 1.44	0.99 0.94	2.07* 2.34	0.94 1.30
Lindsay et al. (2015)^41^	SG: *Lactobacillus salivarius UCC118* (100 mg) PG: no data	84.51 87.40	7.75 10.45	82.35* 82.53	7.57 8.11	13.88 14.61	6.40 9.34	13.04 13.58	5.08 7.73	2.95 3.27	1.42 2.40	3.00 2.85	0.94 1.78
Nabhani et al. (2018)^49^	SG: 500 mg of Lactobacillus probiotic strains consisting of *Lactobacillus acidophilus* (5 × 10^10^ CFU/g), *Lactobacillus plantarum* (1.5 × 10^10^ CFU/g), *Lactobacillus fermentum* (7 × 10^9^ CFU/g), *Lactobacillus gasseri* (2 × 10^10^ CFU/g) and 38.5 mg FOS as prebiotic substance PG: Capsules contained lactose (300 mg), magnesium stearate, talc, colloidal silicon dioxide (5.5 mg each)	90.5 85.8	11.8 10.4	89.2 86.9	11.7 8.6	11.7 12.6	21.6 18.8	11.6 13.5	16.7 16.9	3.2 3.02	2.2 1.7	2.8 3.03	1.9 1.6

*N/A* not available; *SG* supplemented group; *PG* placebo group.

*Significant difference before vs. after (p < 0.05).

The number of individuals in each study ranged from 48 to 149^28,41^. The age of the women ranged from 18 to 45 years. Participants were recruited from the Asian^26–28,38,39,42–46,48,49^ and Caucasian^40, 41^ ethnicities. Studies were designed as randomized controlled trials. Interventions were based on supplementation of probiotic bacteria, such as: *Lactobacillus acidophilus*^26–28,38,42–46^, *Lactobacillus casei*^26,28,42^, *Lactobacillus delbrueckii bulgaricus*^27,43,44^, *Lactobacillus fermentum*^28,45^, *Lactobacillus gasseri*^45^, *Lactobacillus paracasei*^44^, *Lactobacillus plantarum*^44,45^, *Lactobacillus salivarius*^40,41^, *Lactobacillus sporogenes*^39^, *Bifidobacterium animalis*^38,43^, *Bifidobacterium bifidum*^26,28,42,46^, *Bifidobacterium breve*^44^, *Bifidobacterium infantis*^44^, *Bifidobacterium lactis*^27^, *Bifidobacterium longum*^44^, *Streptococcus thermophilus*^27,38,43,44^, as well as synbiotic supplementation, where the prebiotics used were isomalt^39^, sorbitol^39^, inulin^26,48^, and fructo-oligosaccharides^49^.

### Effects of probiotic supplementation on fasting glucose concentration

Initial analysis demonstrated the heterogeneity of the studies (*Q* = 195.73, *p* < 0.0001). There was one outlier: the study of Dolatkhah et al.^43^ The weight of the outlier was the smallest (5.79%) and the studentized residual *t*_*i*_ = − 6.01 was below − 3. The Cook statistic for this study was *D*_*i*_ = 0.88. Although below unity, this value was much higher for Dolatkhah et al.^43^ than for the other studies (where the maximum value was *D*_*i*_ = 0.09). The asymmetry test for the funnel plot was significant (*p* = 0.0001), which indicates the potential presence of publication bias. The trim and fill method showed five missing studies on the left side. The sensitivity test showed that excluding the study of Dolatkhah et al.^43^ decreased the *Q-*statistic to *Q* = 109.52, and had little effect on the result of the meta-analysis (with Dolatkhah et al.:^43^ SMD = − 0.73, 95% CI − 1.39, − 0.08 mg/dl, *p* = 0.0289, *AIC* = 52.59; without Dolatkhah et al.^43^: SMD = − 0.42, 95% CI − 0.75, − 0.09 mg/dl, *p* = 0.0134, *AIC* = 29.34). After excluding Dolatkhah et al.^43^ the asymmetry test was still significant (*p* = 0.0046), but the trim and fill method did not show any missing studies. The AIC criterion was much smaller after excluding Dolatkhah et al.^43^, which means that the model was better adjusted. Moreover, no outliers were found after excluding Dolatkhah et al.^43^ Thus, all analyses of the effects of probiotic supplementation on fasting glucose concentration were done without this study.The average baseline blood glucose concentration ranged from 65.26 ± 22.93 mg/dL to 96.9 ± 7.6 mg/dL in the supplemented groups (SG)^39,45^. Similar results were observed in the placebo groups (PG). Following the intervention, mean fasting glucose concentrations significantly decreased in one study in pregnant women with GDM^38^, and in five studies in pregnant women without GDM^28,40,42,45,46^.

**Figure 2:**
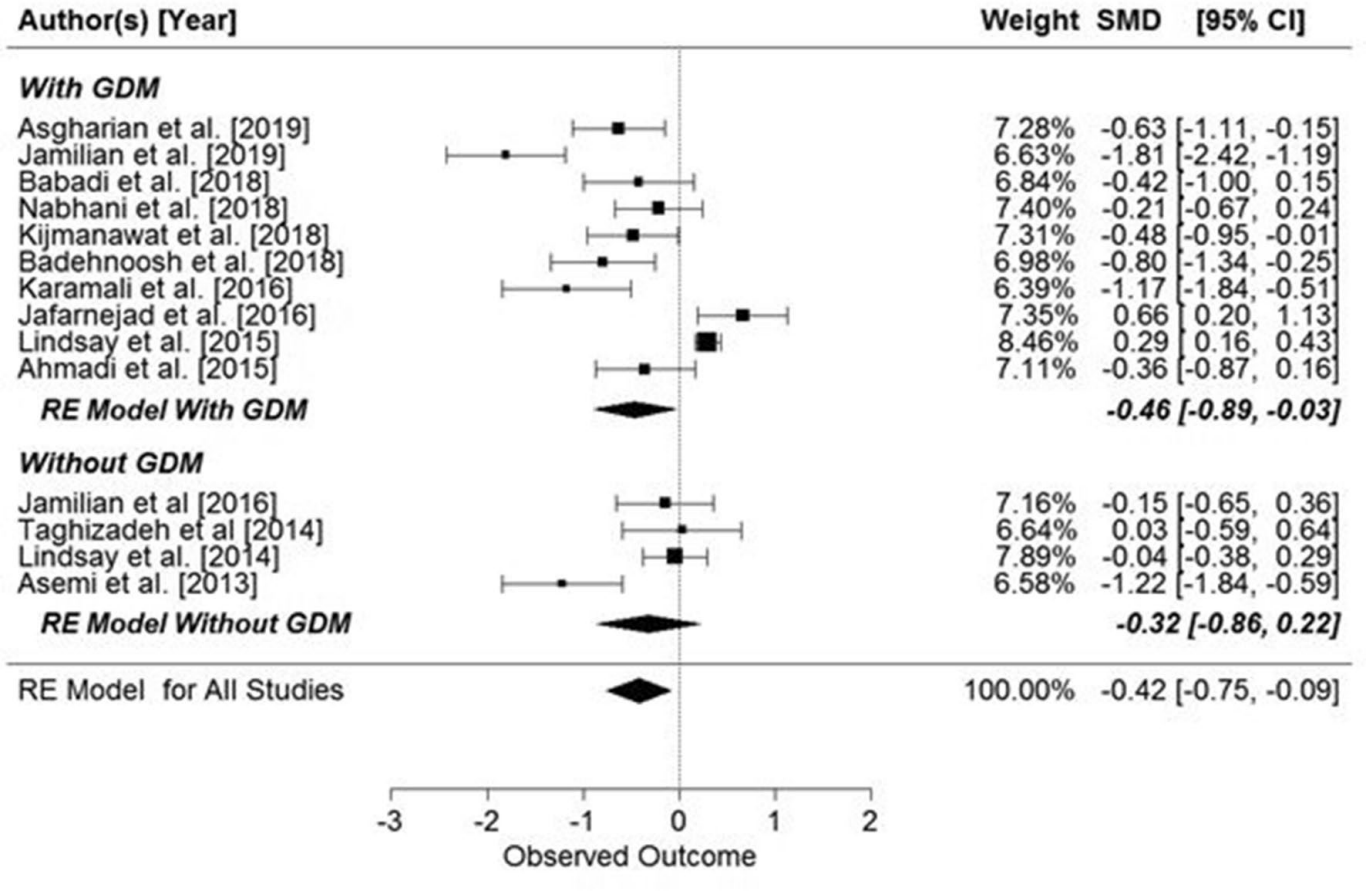
Effect of probiotic and synbiotic supplementation on fasting glucose concentration in women with and without GDM. All studies: SMD = − 0.42, 95% CI − 0.75, − 0.09, p = 0.0134, Z = − 2.47 (p = 0.0134), Q = 109.52 (p < 0.0001), T^2^ = 0.34 (SE = 0.16), df = 13, I^2^ = 88.33%. Women with GDM: SMD = − 0.46, z = − 2.12 (p = 0.034), Q = 94.88 (p = 0.0000), df = 9, T^2^ = 0.41, I^2^ = 90.24%; Women without GDM: SMD = − 0.32, z = − 1.17 (p = 0.2427), Q = 11.48 (p = 0.0094), df = 3, T^2^ = 0.23, I^2^ = 77.80%.

**Figure 3:**
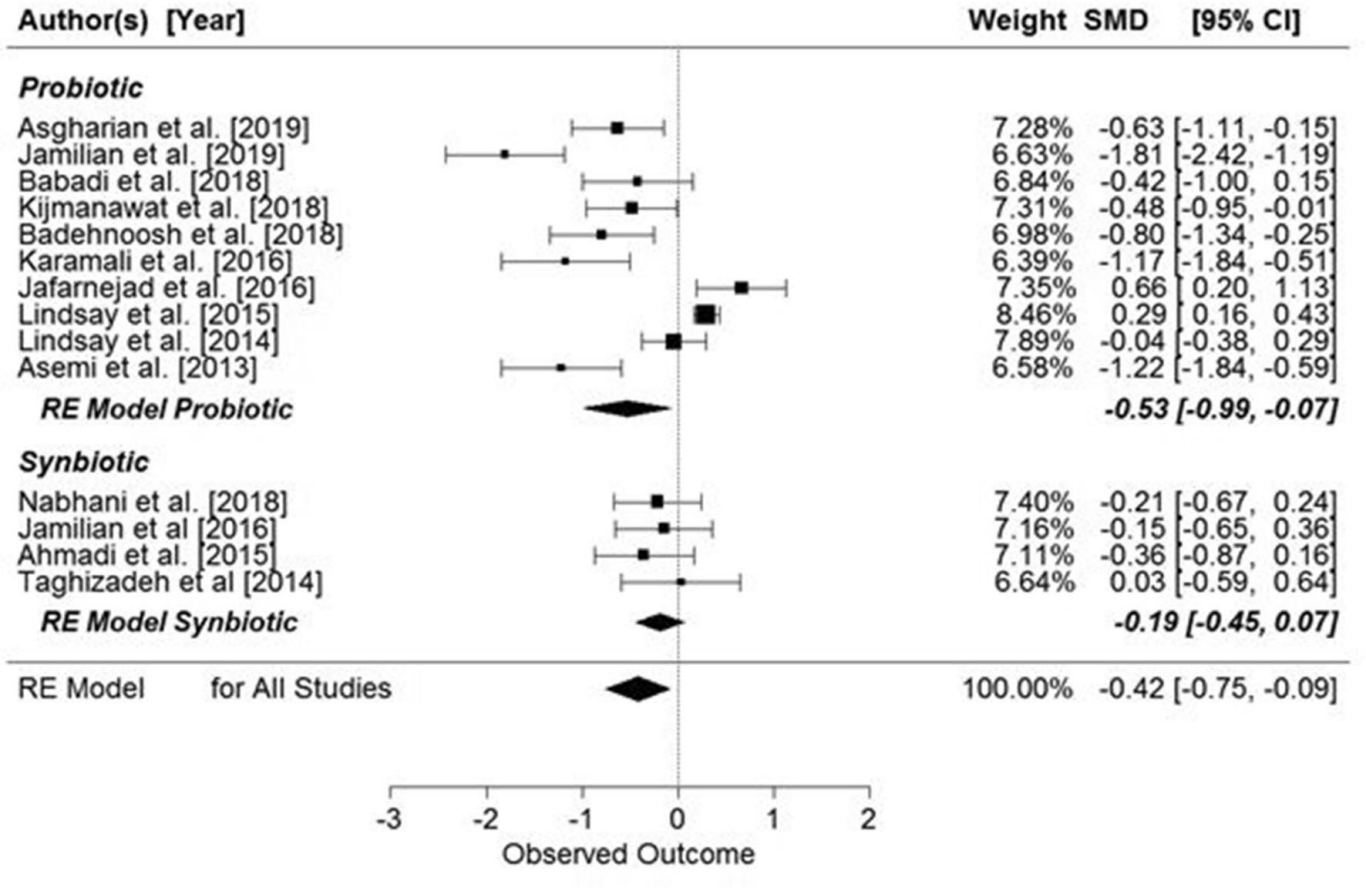
Effects of probiotic or synbiotic supplementation on fasting glucose concentration in pregnant women. Probiotics: SMD = − 0.53, z = − 2.26 (p = 0.02), Q = 106.81 (p < 0.0001); df = 9, T^2^ = 0.48, I^2^ = 92.03%. Synbiotics: SMD = − 0.19, z = − 1.45 (p = 0.15), Q = 0.91 (p = 0.8223), df = 3, T^2^ = 0.00, I^2^ = 0.00%.

The meta-analysis showed significant overall effect of supplementation on fasting glucose concentrations (SMD: − 0.42, 95% CI − 0.75, − 0.09 mg/dl, *p* = 0.0134, Fig. 3), but when the studies were analyzed in two groups, depending on the presence or absence of GDM, supplementation only had a significant effect on pregnant women with GDM (SMD: − 0.46, 95% CI − 0.89, − 0.03 mg/dl, *p* = 0.034, Fig. 2). However, when the data were grouped by supplementation substance (probiotic or synbiotic) significant results were seen only in the probiotic supplemented group (probiotic: SMD: − 0.53, 95% CI − 0.99, − 0.07 mg/dl, *p* = 0.020) (Fig. 3).

### Effects of probiotic supplementation on fasting insulin concentration

Initial analysis demonstrated the heterogeneity of the studies (*Q* = 148.35, p < 0.0001). There was one outlier: the study of Asemi et al.^38^ The weight of this outlier was the smallest (7.15%) and the studentized residual *t*_*i*_ = − 3.36, was below − 3. The Cook statistic for Asemi et al.^38^ was the highest (*D*_*i*_ = 0.56) of all the included studies (the highest *D*_*i*_ among the other studies was 0.29). The asymmetry test for the funnel plot was significant (*p* < 0.0001). The trim and fill method showed three missing studies on the left side. The sensitivity test showed that excluding the study of Asemi et al.^38^ decreased the *Q-*statistic to *Q* = 90.76, and had little effect on the results of meta-analysis (with Asemi et al.^38^: SMD = − 0.93, 95% CI − 1.51, − 0.34 µIU/mL, *p* = 0.0018, *AIC* = 40.41; without Asemi et al.^38^: SMD = − 0.71, 95% CI − 1.14, − 0.27 µIU/mL, *p* = 0.0015, *AIC* = 30.50). After excluding Asemi et al.^38^, one more outlier was found: the study of Dolatkhah et al.^43^ (weight: 6.88%, *t*_*i*_ = − 3.79, *D*_*i*_ = 0.64). There was asymmetry and the trim and fill method showed two missing studies on the left side. Excluding also the study of Dolatkhah et al.^43^ decreased the *Q-*statistic to *Q* = 58.09 and had little effect on the result of meta-analysis (without Dolatkhah et al.^43^: SMD = − 0.53, 95% CI − 0.83, − 0.23 µIU/mL, *p* = 0.0006, *AIC* = 18.67). There were no more outliers after excluding Asemi et al.^38^ and Dolatkhah et al.^43^; the asymmetry test was nonsignificant (*p* = 0.3168) and the trim and fill method showed no missing studies. All analysis of the effects of probiotic supplementation on fasting insulin concentration was thus done without those two studies. Moreover, serum insulin level was not reported in two studies, so the results of eleven studies were considered^26,28,39,40,44–49^. The average baseline fasting insulin concentration ranged from 8.77 ± 5.56 µIU/mL to 19.1 ± 4.2 µIU/mL in the SG^44,46^. Similar results were observed in the PG. Following the intervention mean fasting insulin concentrations decreased in two studies in pregnant women with GDM^39,47^, and in five studies in pregnant women without GDM (Table 4)^26,28,44–46^.

**Figure 4:**
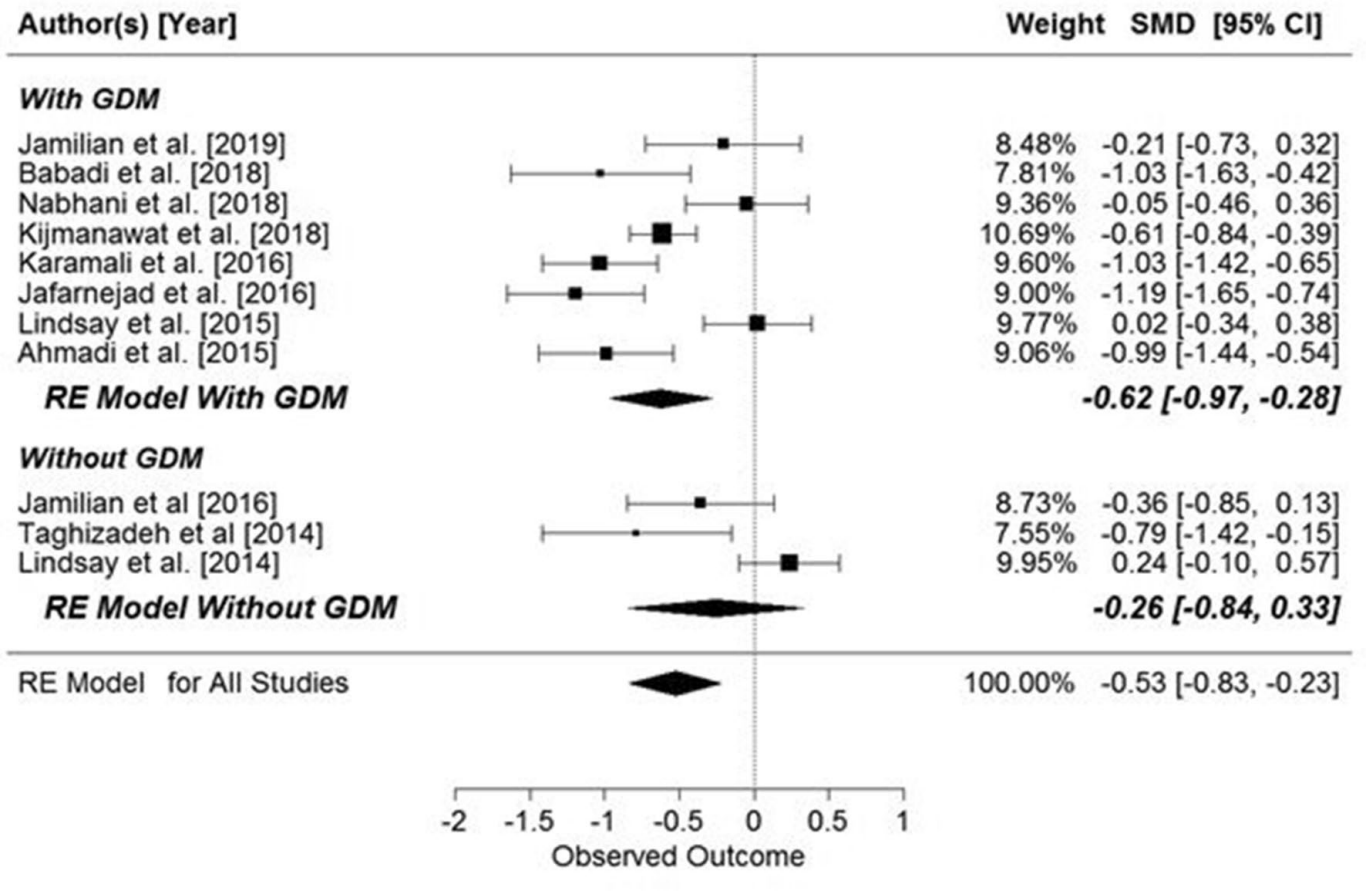
Effect of supplementation on fasting insulin concentration in women with and without GDM. All studies: SMD = − 0.53, 95% CI − 0.83, − 0.23, p = 0.0006, Z = − 3.45 (p = 0.0006), Q = 58.09 (p = 0.0006), T^2^ = 0.2065, df = 10, I^2^ = 83.12%. Women with GDM: SMD = − 0.62, z = − 3.59 (p = 0.0003), Q = 36.67 (p < 0.0001), df = 7, T^2^ = 0.19, I^2^ = 82.94%. Women without GDM: SMD = − 0.26, z = − 0.85 (p = 0.3927), Q = 9.46 (p = 0.0088), df = 2, T^2^ = 0.21, I^2^ = 78.12%.

The meta-analysis showed a significant overall effect of supplementation on fasting insulin concentrations (SMD: − 0.53, 95% CI − 0.83, − 0.23 µIU/mL, *p* = 0.0006) (Fig. 4), but when the studies’ participants were grouped by presence or absence of GDM, supplementation was associated with significantly lowered insulin concentration only in pregnant women with GDM (SMD: − 0.62, 95% CI − 0.97, − 0.28 µIU/mL, *p* = 0.0003) (Fig. 4).

**Figure 5:**
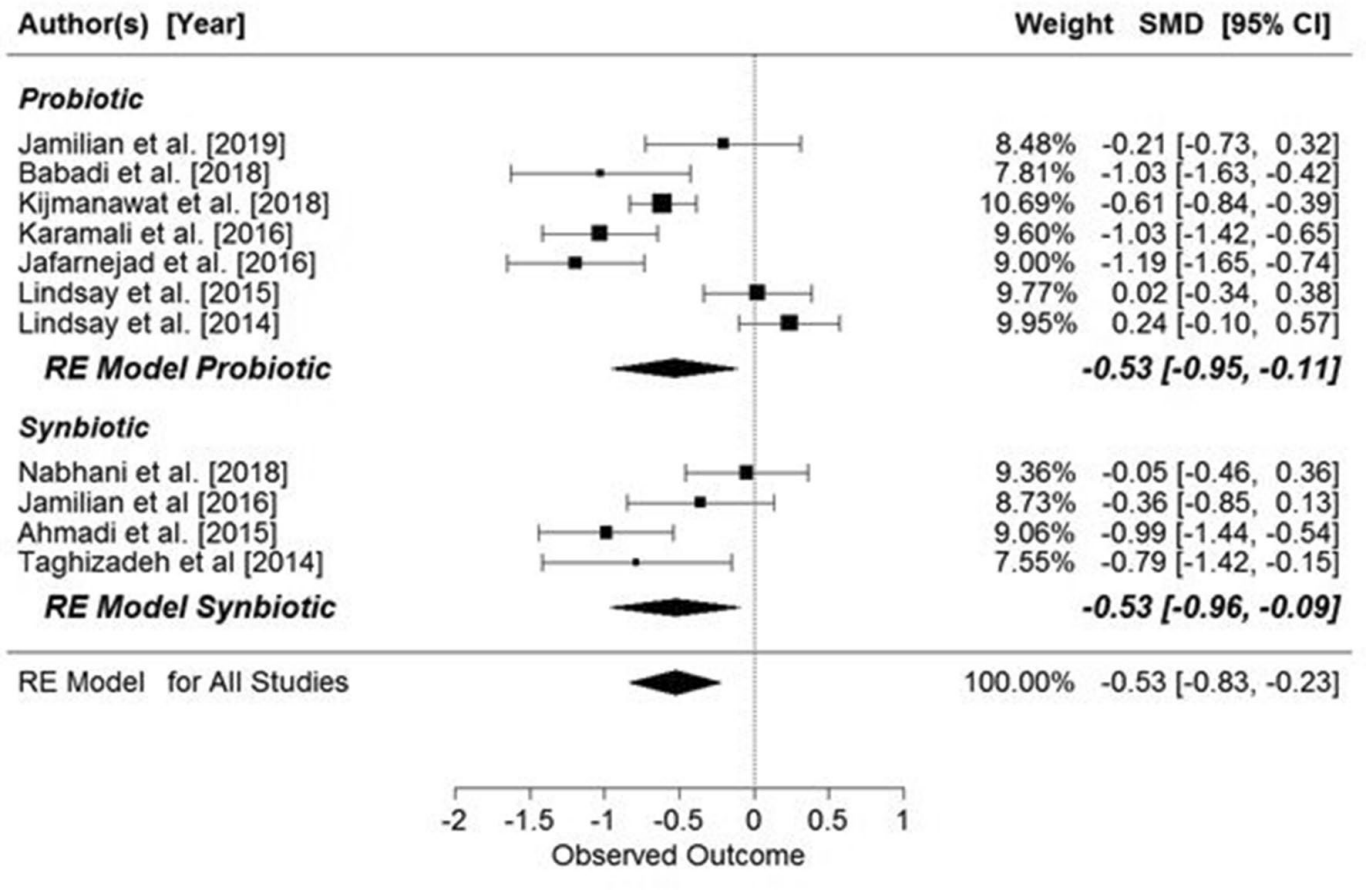
Effects of probiotic or synbiotic supplementation on fasting insulin concentration in pregnant women. Probiotics: SMD = − 0.53, z = − 2.46 (p = 0.0140), Q = 47.88 (p = 0.0000), df = 6, T^2^ = 0.28, I^2^ = 88.51%. Synbiotics: SMD = − 0.53, z = − 2.37 (p = 0.0176), Q = 10.1963 (p = 0.0170), df = 3, T^2^ = 0.13, I^2^ = 68.74%.

When the data were grouped by supplemented substance (probiotic or synbiotic), significant results were seen for both (probiotic: SMD: − 0.53, 95% CI − 0.95, − 0.11 µIU/mL, *p* = 0.0140; synbiotic: SMD: − 0.53, 95% CI − 0.96, − 0.09 µIU/mL, *p* = 0.0176) (Fig. 5).

### Effects of probiotic supplementation on homeostatic model assessment

Initial analysis demonstrated the heterogeneity of the studies (*Q* = 104.08, p < 0.0001). There was one outlier: the study of Dolatkhah et al.^43^ The weight of the outlier was the smallest (6.31%), the studentized residual was *t*_*i*_ = − 5.26, and the Cook statistic was the greatest (*D*_*i*_ = 0.81) of all included studies (the greatest *D*_*i*_ value among the rest of the studies was 0.09). The asymmetry test for the funnel plot was significant (*p* < 0.0001). The trim and fill method showed five missing studies on the left side. The sensitivity test showed that excluding the study of Dolatkhah et al.^43^ decreased the *Q-*statistic to *Q* = 55.33 and had little effect on the result of meta-analysis (with Dolatkhah et al.^43^: SMD = − 0.74, 95% CI − 1.27, − 0.21, *p* = 0.0066, *AIC* = 39.56; without Dolatkhah et al.^43^: SMD = − 0.49, 95% CI − 0.77, − 0.21, *p* = 0.0006, *AIC* = 19.93). After excluding Dolatkhah et al.^43^, the asymmetry test was nonsignificant (*p* = 0.3206), but the trim and fill method found one missing study on the right side. The AIC criterion was much smaller after excluding Dolatkhah et al.^43^, which means that the model was better adjusted. All analysis of the effects of probiotic supplementation on HOMA-IR index were thus performed without that study. Moreover, HOMA-IR values were not available in two studies, so finally results of twelve studies were considered^26,28,38–41,44–49^. In the SG, the mean HOMA-IR ranged from 1.82 ± 0.99 to 4.2 ± 1.2^44,46^. Similar results were observed in the PG (Table 4). Following the intervention, the mean HOMA-IR index decreased in two studies in pregnant women with GDM^39,47^ and in five studies in pregnant women without GDM (Table 4)^26,28,45,46^.

**Figure 6:**
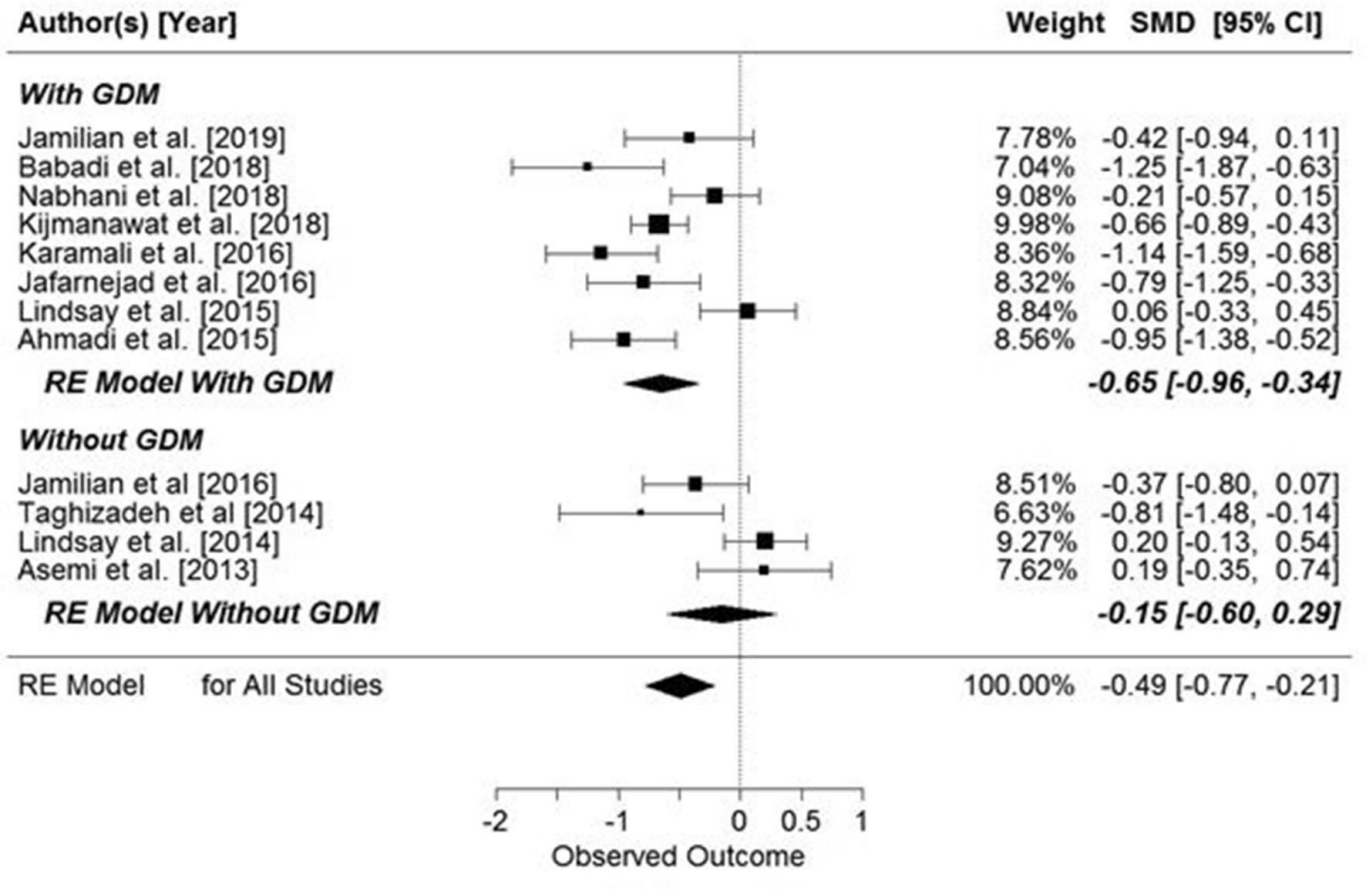
Overall effects of probiotic and synbiotic supplementation on HOMA-IR index in two subgroups of pregnant women, with and without GDM. All studies: SMD = − 0.49, 95% CI − 0.77, − 0.21, p = 0.0066, Z = − 3.45 (p = 0.0006), Q = 55.33 (p = 0.0000), T2 = 0.19, df = 11, I2 = 81.31%. Women with GDM: SMD = − 0.65, z = − 4.10 (p = 0.0043), Q = 28.99 (p = 0.0001), df = 7, T^2^ = 0.15, I^2^ = 78.45%. Women without GDM: SMD − 0.15, z = − 0.68 (p = 0.4997), Q = 9.99 (p = 0.0189), df = 3, T^2^ = 0.14, I^2^ = 71.29%.

**Figure 7:**
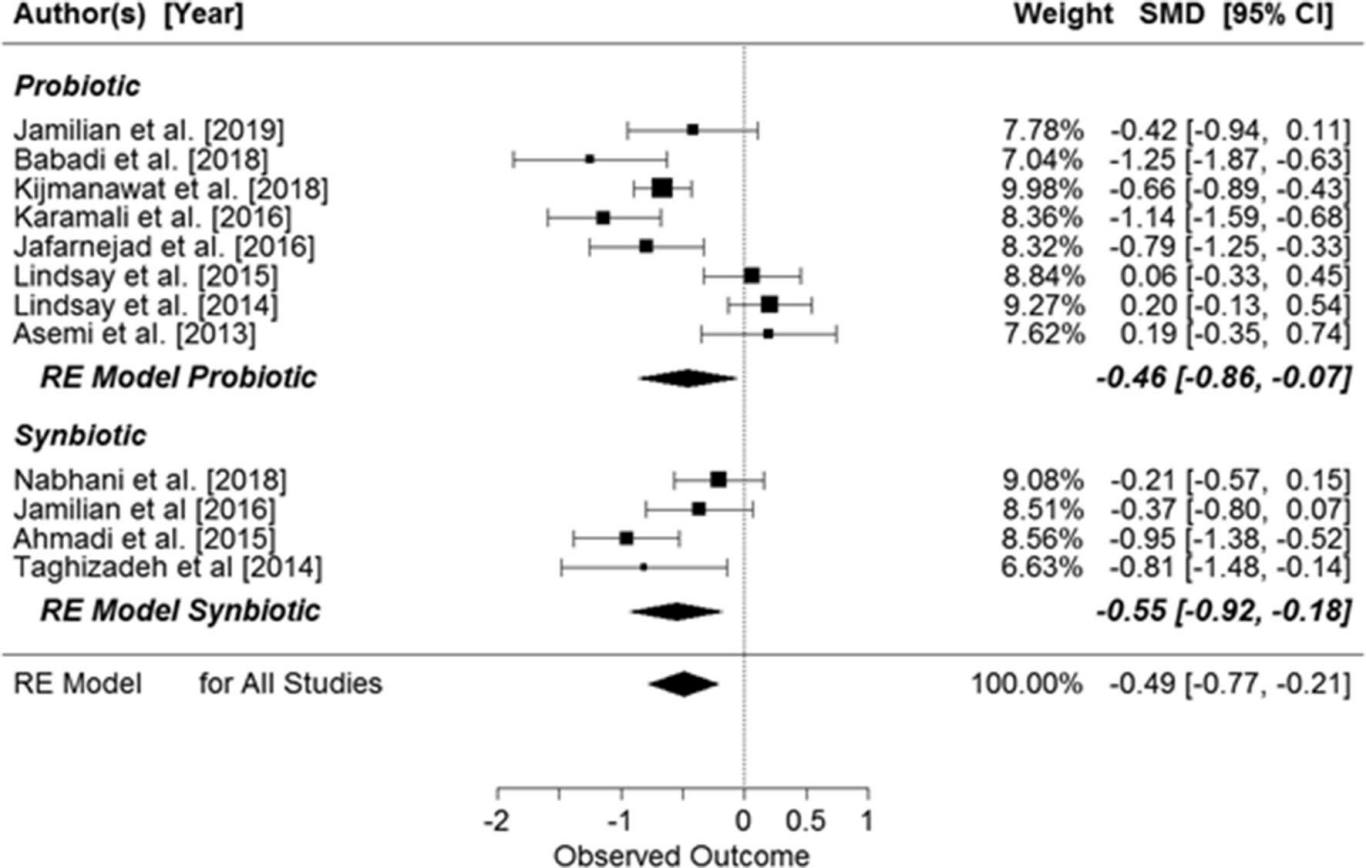
Effects of probiotic or synbiotic supplementation on HOMA-IR index. Probiotics: SMD − 0.46, z = − 2.2953 (p = 0.0217), Q = 47.06 (p = 0.0000), df = 7, T^2^ = 0.27, I^2^ = 86.54%; Synbiotics: SMD − 0.55, z = − 2.94 (p = 0.0033), Q = 8.02 (p = 0.0456), df = 3, T^2^ = 0.09, I^2^ = 61.79%.

There was significant effect of supplementation on the HOMA index in pregnant women (SMD: − 0.49, 95% CI − 0.77, − 0.21; p = 0.0066, Fig. 6), but when the results were compared for the those with and without GDM, the significant effects of supplementation were observed only in women with GDM (pregnant women with GDM: SMD: − 0.65, 95% CI − 0.96, − 0.34, *p* = 0.0043) (Fig. 6). Further, when the studies were grouped by the supplemented substance (probiotic or synbiotic), significant results were seen in both groups (probiotic: SMD: −0.46, 95% CI − 0.86, − 0.07, *p* = 0.0217; synbiotic: SMD: − 0.55, 95% CI − 0.92, − 0.18, *p* = 0.0033) (Fig. 7).

## Discussion

In this study, we showed that the intake of supplements containing probiotics or synbiotics positively affects the carbohydrate metabolism in pregnant women, but that this effect depends on the type of supplement (probiotic or synbiotic) and on the presence or absence of GDM. Our analysis showed that such supplementation lowers serum glucose levels, insulin levels, and HOMA-IR index, but that the effect was only significant in pregnant women with GDM. Moreover, both probiotics and synbiotics lowered insulin serum level and HOMA-IR index, but the glucose-lowering effect was specific to probiotics. Our results indicate that this kind of supplementation may help improve the glucose metabolism of pregnant women, but only those diagnosed with gestational diabetes. Further, the addition of prebiotics to probiotics does not increase this effect in the case of serum glucose or insulin concentration.

The similar meta-analysis previously performed by Peng et al.^25^ showed opposite results, namely that probiotic supplementation positively affected glycemic control, but only in pregnant women without GDM. The discrepancy is mainly the result of the different studies included in the meta-analyses, and also the different method of statistical analysis. We did not include the study of Laitinen et al.^50^, because it tested the cumulative effect of probiotics and diet, which was outside the scope of our analysis. We also did not include the Wickens et al.^51^, because of insufficient data: these studies lacked information on fasting glucose levels and mean and standard deviation of the parameters. From the same reasons, studies of Oksene-Gafa et al.^52^, Callaway^53^, were also excluded. Moreover, we also excluded the work of Dolatkhah et al.^43^ from our analysis, because it was the outlier for all parameters. In our analysis, we additionally deliberated on the differences between probiotic and synbiotic effect on glucose metabolism, while Peng et al.^25^ synbiotics were treated equally with probiotics. We additionally estimated the effect size using the pretest–posttest–control method, whereas Peng et al.^25^ only examined postintervention results.

A positive effect of probiotic supplementation on glycemic control has been shown in other studies. A recent meta-analysis of 17 RCTs which included adults with and without hyperglycemia revealed that probiotic supplementation lowers serum glucose, insulin, and HOMA-IR; this effect was greater in hyperglycemic subjects^54^. Similar results were found in our meta-analysis, showing that women with GDM benefit more than women without GDM. This may result from the fact that those with GDM or hyperglycemia may have more disrupted microbiota composition, which probiotic supplementation can restore^55^. By contrast, people lacking problems with glucose metabolism may have healthy gut microbiota composition and probiotic supplementation does not alter this state much. The differences in gut microbiota composition between people with and without type-2 diabetes mellitus (T2DM) have been indicated to play a role in the development and control of this disease. It has been reported that a high prevalence of insulin resistance is correlated with low gut microbiota diversity, which entails proinflammatory properties. It has been suggested that these properties are caused by a reduction in the amount of butyrate-producing bacteria and an increase in mucin-degrading bacteria. These changes may impair the gut integrity through endotoxemia, which causes low-grade inflammation. In endotoxemia, elevated plasma levels of lipopolysaccharides (LPS) impair glucose metabolism. Moreover, lower concentrations of SCFAs, such as butyrate, may lead to a decrease in the intestinal expression of genes encoding satiety hormones, such as peptide YY and glucagon-like peptide^55^. The bacteria that are less abundant in subjects with T2DM belong to the phylum *Firmicutes*, and the most prominent decline is observed for *Roseburia* and *Faecalibacterium prausnitzii*^3,56^. These bacteria are known as human gut colonizers and butyrate producers, and have been reported to improve diabetic control and insulin sensitivity. The different composition of the community of gut microbiota in people with T2DM is associated with changes in the metabolic capacity of this community. The pathways that are more abundant in T2DM subjects are for starch and simple sugar metabolism. This supports the concept of an increased capacity for energy harvest in people with T2DM, which can lead to obesity^3^.

In our meta-analysis, we examined the impact of probiotic supplementation on glucose metabolism in pregnant women and determined whether GDM has an effect on the effectiveness of such interventions. Although, as mentioned above, a positive effect of probiotic supplementation has been demonstrated in people with T2DM, GDM is not the same condition as T2DM. First of all, pregnancy alters the metabolism. Secondly, the duration of disrupted glucose metabolism in women with GDM is short, comparing to subjects with T2DM taking part in the studies, where a disrupted glucose metabolism may have existed for many years. This is important given that gut microbiota composition may change over time and is different in prediabetic and diabetic people. It has been shown that a total of 28 operational taxonomic units (OTUs) are related to T2DM status and that the relative abundances of *Bacteroides* and *Clostridium* undergo marked changes with progression of the disease^57^. Taking this into consideration, it is possible that different probiotics may be needed to restore proper microbiota composition in those with prediabetes and in those with T2DM or GDM. As the microbiota of people with T2DM is more disrupted, they may be more responsive to manipulations of the gut microbiota aimed at improving glucose metabolism^57^.

The effect of microbiota on glucose metabolism in pregnant women, which this meta-analysis has focused on, may be different than in non-pregnant women. This is because the gut microbiota of pregnant women (especially in the third trimester) and nonpregnant women differs, and because pregnancy implies some adaptations in the metabolism, such as insulin resistance, in order to facilitate fetal growth and development. Koren et al.^58^ showed that the composition of gut microbiota changes dramatically from first to third trimester. For example, *Faecalibacterium* are less abundant and *Proteobacteria* and *Actinobacteria* are enriched in women in third trimester. Moreover, a loss of microbiota richness is observed. Such changes predispose to inflammation-associated dysbiosis. Dysbiosis, inflammation, and weight gain characterize metabolic syndrome, and are generally considered to negatively affect health; however, they seem to be normal or even beneficial in pregnancy, as they promote energy storage in fat tissue and provide for the growth of the fetus^59^. Taking this into account—as well as the results of this meta-analysis, which showed that probiotic supplementation does not have a significant impact on glucose metabolism of pregnant women without GDM—it seems that such attempts aimed at lowering glucose and insulin concentration in women without GDM are pointless. However, it should be added that this may be due to the insufficient number of studies included in the analysis, as there were only four studies with women without GDM. It should also be noted that an additional determinant of the effectiveness of probiotic therapy in preventing GDM in pregnant women, which may be worthy of analysis, is the assessment of the frequency of GDM development in the supplemented population. Such a procedure would also make it possible to conclude whether insulin and fasting glucose levels, the HOMA index, or the frequency of GDM development are better indicators of the effectiveness of probiotic therapy in pregnant women. Unfortunately, this was not possible with the studies considered there, because only Lindsay et al. gave data on the frequency of GDM development in the study group—this was 16.1% in the supplemented group and 14.9% in the placebo group.

The beneficial effects of probiotic supplementation in pregnant women with GDM may arise from the fact that, according to Crusell et al.^59^, the gut microbiota composition of those women differs from that of pregnant women without GDM. Although the diversity of microbiota is similar, these authors identified seventeen species-level OTUs, predominantly within the phylum *Firmicutes*, which were differentially abundant in women with and without GDM in the third trimester. They suggested some genera as biomarkers of GDM, including *Collinsella*, *Rothia*, *Actinomyces*, *Desulfovibrio*, *Leuconostoc*, and *Mogibacterium.* This study also shows that other disturbances appear in GDM which differed from those in T2DM (mentioned earlier). It can thus be assumed that probiotic therapies that working on T2DM subjects may not work on GDM women, and vice-versa. This shows the importance of such studies.

Interestingly, we have found that adding prebiotics to probiotics as a synbiotic is no more beneficial in lowering glucose concentration than probiotics alone. For insulin, the effect was the same as for probiotics, while for HOMA-IR the effect was slightly better than for solely probiotic supplementation. Prebiotics, such as inulin or fructooligosaccharides (FOS), are substrates with which probiotic bacteria can produce SCFA. Most human intervention studies have shown a positive effect of synbiotic supplementation on glucose metabolism^60,61^. However, few studies have compared the effects of probiotic and synbiotic supplementation. One such study was performed by Kassaian et al.^62^, and showed that in prediabetic adults, both synbiotics and probiotics lower plasma glucose, insulin, and HOMA-IR. However, the effect of synbiotics was not better than that of probiotics alone. In two studies included in this meta-analysis^39,49^, the probiotic part of the synbiotic included different strains of *Lactobacillus*, whereas another two studies^26,47^ used both *Lactobacillus* and *Bifidobacterium*. It was found that not all strains of *Lactobacillus* and *Bifidobacterium* have the ability to ferment FOS and inulin, and that the metabolic potential to ferment those prebiotics may differ between various strains of these probiotic bacteria^63,64^. Those results may partly explain the results of our meta-analysis, and this emphasizes the importance of the proper selection of probiotics and prebiotic, and their combination as synbiotic supplements.

The results of some individual studies^41,47^ show that probiotic supplementation does not improve glucose metabolism. This may be due to the different formulations of probiotics and synbiotics used in those studies. Some studies used only *Lactobacillus* species, while others employed combinations of *Lactobacillus*, *Bifidobacterium*, and *Streptococcus*^39–41,49^. There are studies indicating that multistrain probiotics appear to show better efficacy than single strains in treating various conditions^65^. The duration of the supplementation also seems to be important: in the studies included in this meta-analysis, the duration of intervention lasted from four to twelve weeks. The observed effects may be dose-dependent^66^, which may also explain the differences seen in the results of the studies, where the dose varied from 10^6^ to over 10^10^ CFU per capsule; however, some studies did not indicate the dose of bacteria used.

The strength of our meta-analysis is that the studies included are characterized by similar groups of people (pregnant adult women) and that the only experimental factor tested was the use of probiotic or synbiotic supplementation. Moreover, our statistical analysis considered both the presupplementation and postsupplementation concentration in the placebo and intervention groups, whereas many analyses only make use of the postintervention concentrations. The limitations of our study include not considering the dose, duration, or week of pregnancy at the start of supplementation. Most of the studies used different doses and different species of probiotic bacteria, which made it impossible to include these factors in the meta-analysis. Furthermore, most included samples consisted of relatively small groups, typically of 30–40 women. The results of the individual studies may also have been affected by many other factors that were not taken into account. The mother’s periconceptional diet is an example of a factor that could affect the effectiveness of microbiota modulation^67^.

## Conclusions

In conclusion, probiotic supplementation may improve glucose metabolism in pregnant women, especially in those with GDM. There is a need for more randomized controlled trials of women with and without GDM, with larger sample sizes, in order to better determine this effect. Moreover, it is necessary to determine the best timing, duration, composition, and dose of such supplementation. Dietary intake, physical activity, and baseline gut microbiota composition should also be examined in such studies, as the effectiveness of probiotic supplementation may depend on these factors.
